# Supplementary material for: Machine learning–based observation-constrained projections reveal elevated global socioeconomic risks from wildfire
Source: Nat Commun. 2022 Mar 22;13:1250. doi: 10.1038/s41467-022-28853-0 (PMC8940959; doi:10.1038/s41467-022-28853-0)
Supplement: Supplementary file 1 — Supplementary Information File [file 41467_2022_28853_MOESM1_ESM.pdf]

Supplementary information for:
Machine learning–based observation-constrained projections reveal elevated global socioeconomic risks from wildfire

Yan Yu[1, †], Jiafu Mao[2,*, †], Stan D. Wullschleger[2], Anping Chen[3], Xiaoying Shi[2], Yaoping Wang[4], Forrest M. Hoffman[5], Yulong Zhang[4], and Eric Pierce[2]

[1]*Department of Atmospheric and Oceanic Sciences, School of Physics, Peking University, Beijing, China*
[2]*Environmental Sciences Division and Climate Change Science Institute, Oak Ridge National Laboratory, Oak Ridge, TN, USA*
[3]*Department of Biology and Graduate Degree Program in Ecology, Colorado State University, Fort Collins, CO, USA*
[4]*Institute for a Secure & Sustainable Environment, University of Tennessee, Knoxville, TN, USA*
[5]*Computational Sciences and Engineering Division and Climate Change Science Institute, Oak Ridge National Laboratory, Oak Ridge, TN, USA*

[*]Jiafu Mao: *Environmental Sciences Division and Climate Change Science Institute, Oak Ridge National Laboratory, Oak Ridge, TN 37830; +1 865-576-7815; maoj@ornl.gov*
[†]These authors contributed equally to this work.

Content of this file:
Supplementary Tables 1-3
Supplementary Figures 1-14

Supplementary Table 1 List of CMIP6 ESMs in this analysis of fire carbon emission.

Model	Nominal resolution (km)	Number of ensemble members	Land model	Fire model description
CESM2[1]	100	3	CLM5	Natural and anthropogenic ignition sources and suppression of agricultural, deforestation, and peat fires[2,3]
CESM2-WACCM[1]	100	5		
CMCC-ESM2[4]	100	1	CLM4.5	
CNRM-ESM2.1[5]	250	5	SURFEXv8.0 (ISBA)	Interactive natural fires[5]
E3SM-1.1[6]	100	1	ELM v1.1	Same as CLM5
EC-Earth3-CC[7]	100	1	LPJ-GUESS v4	Interactive natural fires[7]
EC-Earth3-Veg-LR[7]	250	3		
EC-Earth3-Veg[7]	100	5		
GFDL-ESM4[8]	100	1	LM4.1	Distinct parameterizations for natural and agricultural wildfires, especially representing multiday and crown wildfires[9]
MPI-ESM1-2-LR[10]	250	10	JSBACH3.20	Natural fires ignited by human activity and lightning, with up to 12 h duration[10]
MRI-ESM2[11]	100	1	HAL 1.0	N/A
NorESM2-LM[12]	250	1	CLM5	Same as CLM5
NorESM2-MM[12]	100	1		

Supplementary Table 2 Summary of the observational, reanalysis, and remote sensing data sets applied in the observational constraint. Select references on each constraining variable are listed.

**Variable**	**Data Set**	**Period**	**Spatial Resolution**
Fire carbon emission	Global Fire Emissions Database[13]	1997–2016	0.25°×0.25°
	The European Centre for Medium-Range Weather Forecasts Global Fire Assimilation System[14]	2003–2020	0.1°×0.1°
Leaf area index (LAI)	Global Inventory Monitoring and Modeling System third-generation LAI[15]	1982–2015	0.0833°×0.0833°
	Long-term Global Mapping LAI[16]	1982–2018	0.0833°×0.0833°
	National Oceanic and Atmospheric Administration Climate Data Record of AVHRR LAI[17]	1982–2020	0.05°×0.05°
Surface-layer soil moisture	Global Land Evaporation Amsterdam Model v3.3a[18]	1980–2018	0.25°×0.25°
	European Centre for Medium-Range Weather Forecast Reanalysis version 5 (ERA-5)[19]	1980–2020	0.25°×0.25°
	Earth System Model-independent merged soil moisture product[20]	1970–2016	0.5°×0.5°
2 m air temperature[21,22]	University of Delaware (UDEL) Terrestrial precipitation v5.01[23]	1900–2017	0.5°×0.5°
	University of East Anglia Climatic Research Unit (CRU) high resolution gridded data TS4.03[24]	1901–2018	0.5°×0.5°
Precipitation[21,22]	UDEL v5.01[25]	1900–2017	0.5°×0.5°
	CRU TS4.03[24]	1901–2018	0.5°×0.5°
	Global Precipitation Climatology Centre v2018[26]	1891–2016	0.5°×0.5°
2 m wind speed and relative humidity[22]	National Aeronautics and Space Administration Modern-Era Retrospective Analysis for Research and Applications v2[27]	1980–2019	0.625°×0.5°
	ERA-5[28]	1980–2019	0.25°×0.25°
Monthly flash rate[29]	Lightning Imaging Sensor on the Tropical Rainfall Measurement Mission[30]	1997–2015	0.5°×0.5°
Orography[31–33]	TerrainBase Global 5 arc-minute ocean depth and land elevation from the United State National Geophysical Data Center[34]	1995	5 arc-min
Annual land use statistics, including fractions of forested primary land, managed pasture, rangeland, cropland, and urban land[21]	Land use harmonization[35]	850–2100	0.25°×0.25°

Population density[21]	History Database of the Global Environment[36]	10,000 BCE to 2017 CE	0.0833°×0.0833°

Supplementary Table 3 Top 10 countries projected to experience greatest relative changes in socioeconomic risks caused by wildfires in the twenty-first century, according to the default and observation-constrained multimodel ensemble. The relative trend (Rel. trend) is calculated as the ratio of the projected trend and the corresponding socioeconomic risk during the 2010s. African countries are represented by orange shading, Asian countries by yellow shading, and European countries by blue shading.

#	**Population risk (10^3 kg m^{-2} y^{-1} person)**			**Gross domestic product risk (kg m^{-2} y^{-1} billion USD)**			**Agricultural risk (kg m^{-2} y^{-1} km^2)**		
	Country	Trend (dec^{-1})	Rel. trend (% dec^{-1})	Country	Trend (dec^{-1})	Rel. trend (% dec^{-1})	Country	Trend (dec^{-1})	Rel. trend (% dec^{-1})
Original ensemble									
1	Bahrain	1.0	260	Djibouti	0.265	3,932	Yemen	18.3	97
2	Djibouti	4.8	200	Niger	0.781	2,111	Namibia	313.6	75
3	Yemen	34.3	110	Qatar	0.293	2,102	Norway	24.2	71
4	United Arab Emirates	11.9	90	Eritrea	0.270	1,644	Botswana	489.3	70
5	Niger	35.6	85	Somalia	0.156	1,629	Chad	529.9	67
6	Kuwait	2.2	70	Yemen	0.630	1,029	Sweden	210.4	59
7	Eritrea	20.1	70	Sudan	1.678	755	Sudan	1,753.9	57
8	Oman	3.9	60	Malawi	2.142	641	Switzerland	12.8	55
9	Luxemburg	3.4	66	Zambia	8.058	637	Eritrea	33.2	51
10	Ireland	5.3	60	Tajikistan	0.831	597	Oman	1.2	51
Observation-constrained ensemble									
1	Niger	58.7	141	Niger	0.778	2,102	Liberia	180.5	192
2	Uganda	402.1	108	Sierra Leone	1.065	2,070	Sierra Leone	284.6	171
3	Ireland	7.1	84	Uganda	5.695	2,009	Ireland	11.6	95
4	Nigeria	1,410.5	78	Malawi	4.173	1,249	Guinea	718.1	81
5	Yemen	22.0	72	Benin	2.391	1,233	Senegal	246.9	76
6	Sierra Leone	78.1	66	Democratic Republic of the Congo	13.028	1,227	Chad	586.8	74
7	Pakistan	348.0	56	Nigeria	36.966	1,112	Congo	110.8	74
8	Malawi	236.5	52	Yemen	0.641	1,046	Yemen	13.7	73
9	Benin	99.2	53	Guinea	2.504	991	Sudan	1,953.3	63
10	Democratic Republic of Congo	1,326.9	46	Burundi	0.487	789	The Gambia	21.4	58

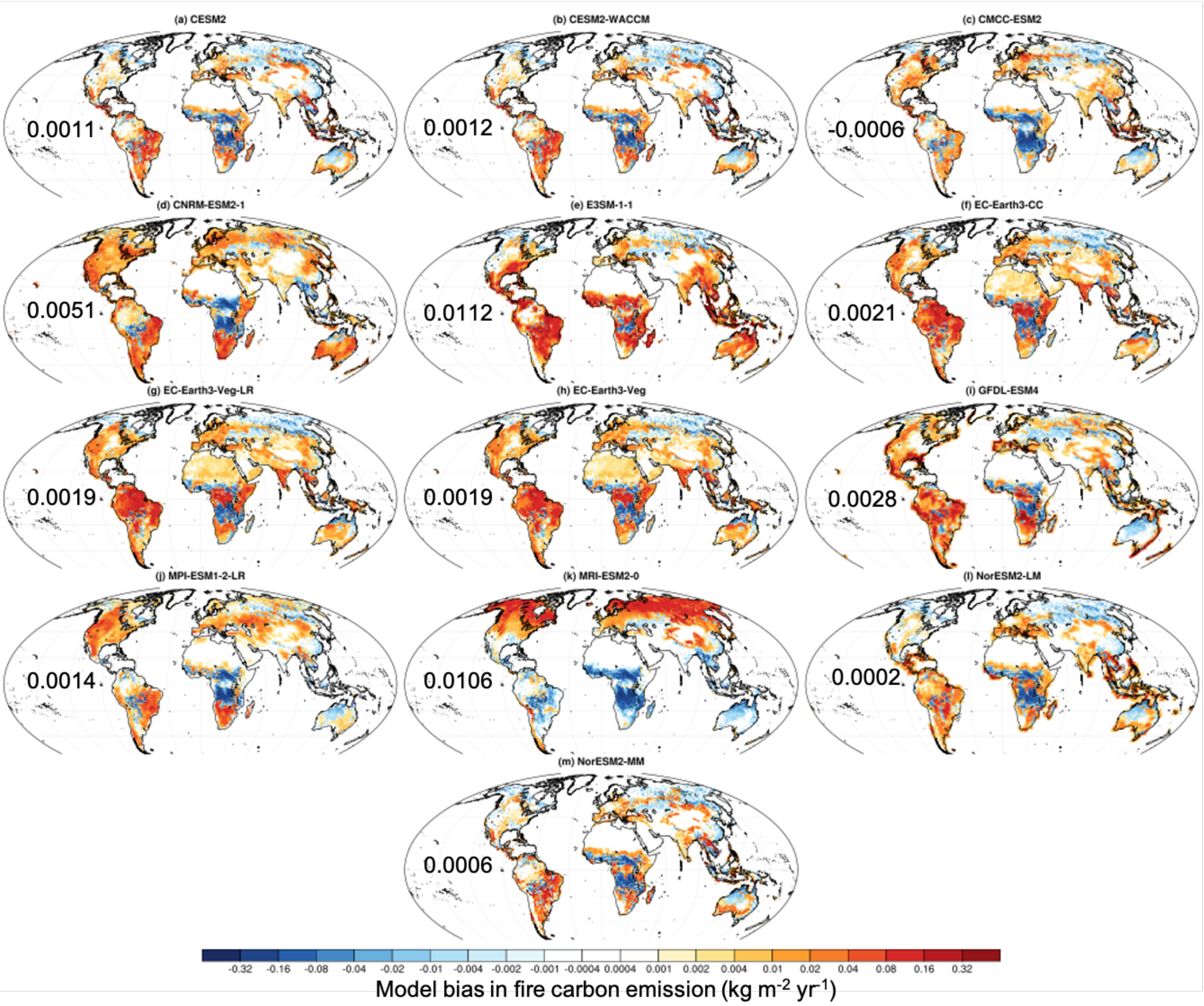

Supplementary Fig. 1: Mean model bias in historical fire carbon emission during 2007–2016 from the examined 13 Earth system models (ESMs). The mean bias for each model is obtained by first subtracting fire carbon emission (kg m^{-2} yr^{-1}) reported by each ensemble member with that from two observational data sets, and then averaging across different ensemble members and observational data sets. The global mean bias of each model is denoted in the corresponding panel.

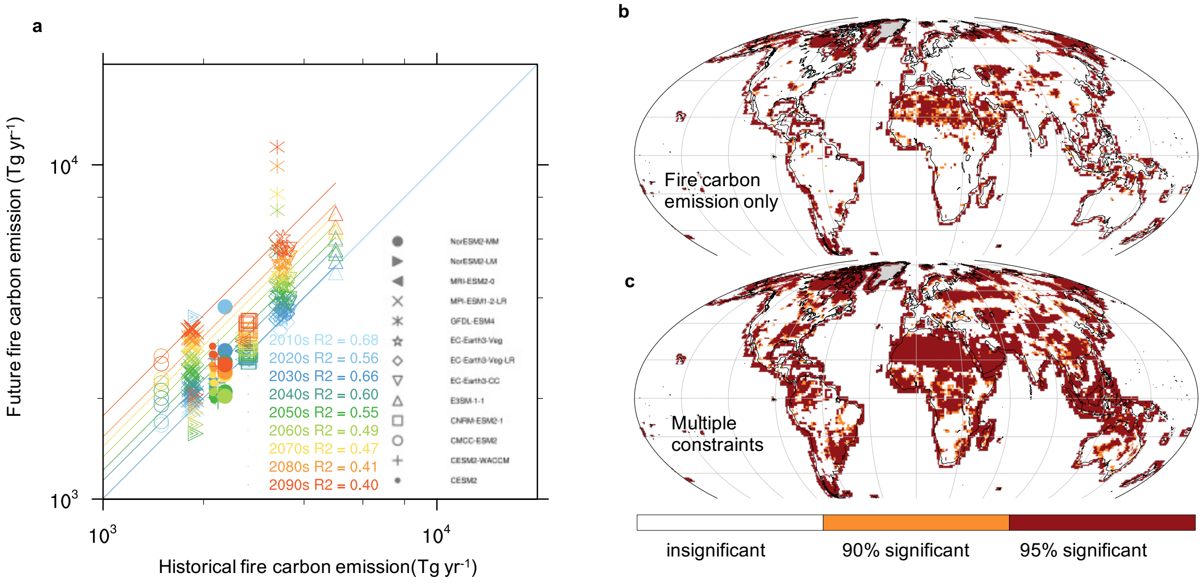

Supplementary Fig. 2: Limited applicability of traditional emergent constraint (EC) in the projection of fire carbon emission with CMIP6 models. a. Scatterplot of global total future fire carbon emission (Tg yr^{-1}) during the 2010s, 2020s, …, and 2090s versus historical fire carbon emission (Tg yr^{-1}) during the 2000s among 13 CMIP6 ESMs and their ensemble members. The squared correlation coefficient (R^2) between future and historical fire carbon emission for each future decade is indicated in the legend. These R^2s are all significant at the $p < 0.05$ level ($n = 38$). **b.** Level of significance of the cross-model correlation between the historical and future fire carbon emission at each grid cell. **c.** Level of significance of the cross-model correlation between the optimized linear combination of multiple historical constraining variables and future fire carbon emission at each grid cell. The constraining variables in **c** include historical fire carbon emission, leaf area index, soil moisture, temperature, precipitation, relative humidity, and wind speed. In **b** and **c**, the correlation is first obtained for each future decade and then averaged across different future decades. The significance of the multidecade average correlation is evaluated using the two-tail Student's t-test. Coastal areas mostly show significant correlation between future fire carbon emission and constraining variables because of minimal wildfire activity from these regions in both historical and future simulations.

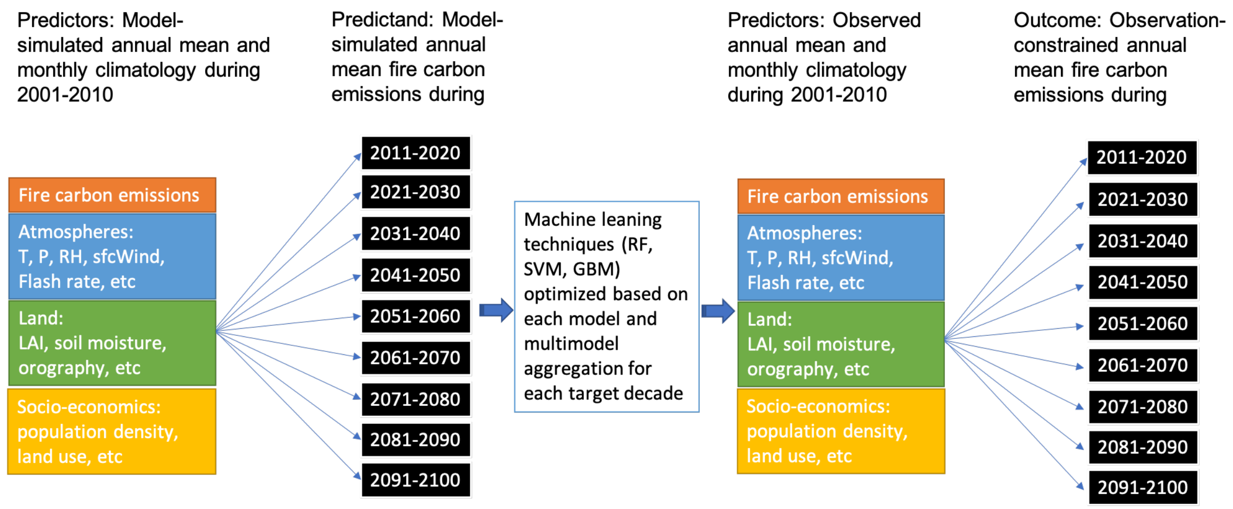

Supplementary Fig. 3: Schematic of the current analytical framework.

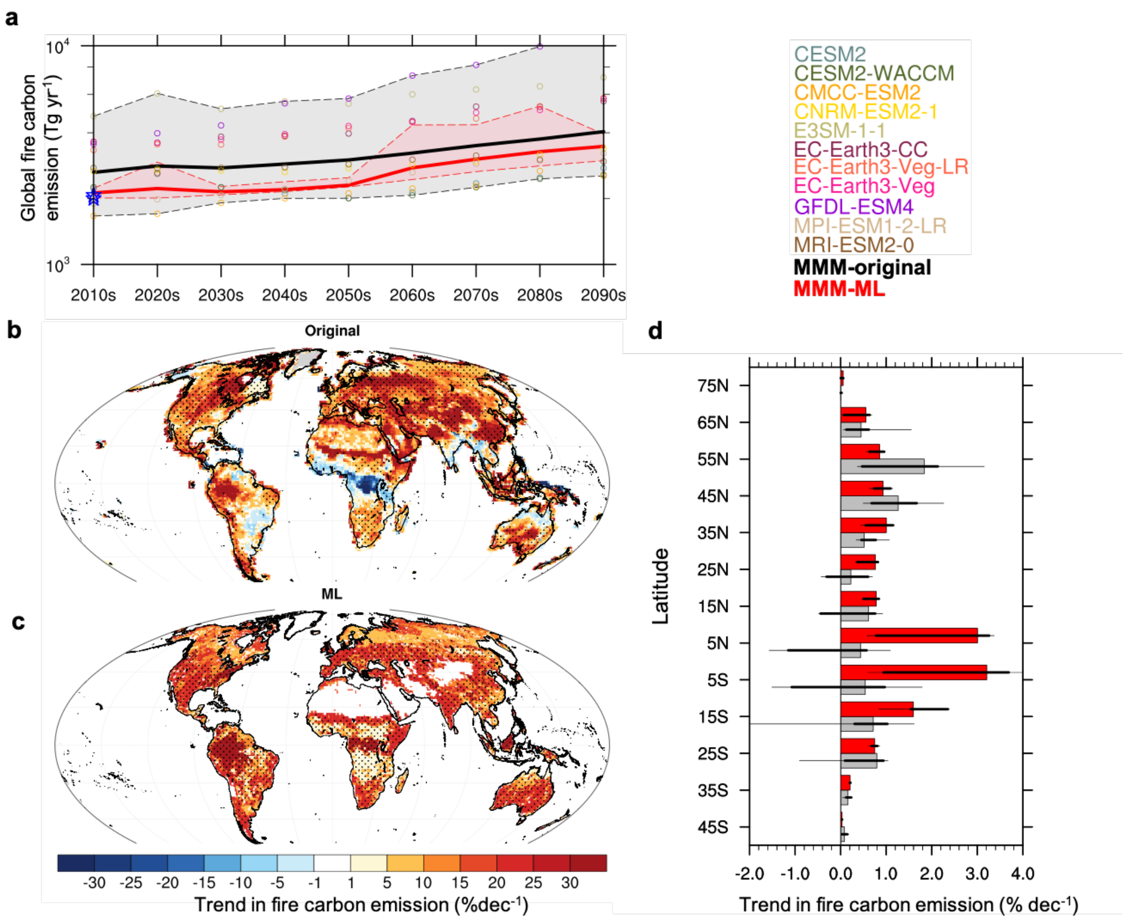

Supplementary Fig. 4: Global and latitudinal evolution of fire carbon emissions from the original and observation-constrained multimodel ensembles, excluding the NorESM models. a. Time series of global total fire carbon emission (Tg yr^{-1}) from the 2010s to 2090s, according to the original individual ESMs (circles), their multimodel mean (black thick line), and the observation-constrained multimodel, multi–data set mean (red thick line). The pink shading with dashed boundaries indicates the 10th and 90th percentiles in the multimodel, multi–data set observation-constrained ensemble; the gray shading with dashed boundaries indicates the 10th and 90th percentiles in the original multimodel ensemble. The blue stars indicate the observed global fire carbon emission from two data sets. **b.** Unconstrained and **c.** constrained multimodel mean trend in fire carbon emission (change per decade as the percentage of the historical fire carbon emission in the 2010s) from the 2010s to 2090s. Stitches indicate areas with a robust trend in fire carbon emission, with a consistent sign of trend among at least 80% of the ensemble members. **d.** Trend in the total fire carbon emission (change per decade as the percentage of the historical fire carbon emission in the 2010s) from each 10° zonal band during the 2010s to 2090s, according to the original (multimodel mean: gray bars; 25th–75th percentiles: thick horizontal lines; 10th–90th percentiles: thin horizontal lines) and observation-constrained (multimodel mean: red bars; 25th–75th percentiles: thick horizontal lines; 10th–90th percentiles: thin horizontal lines) ESM simulations.

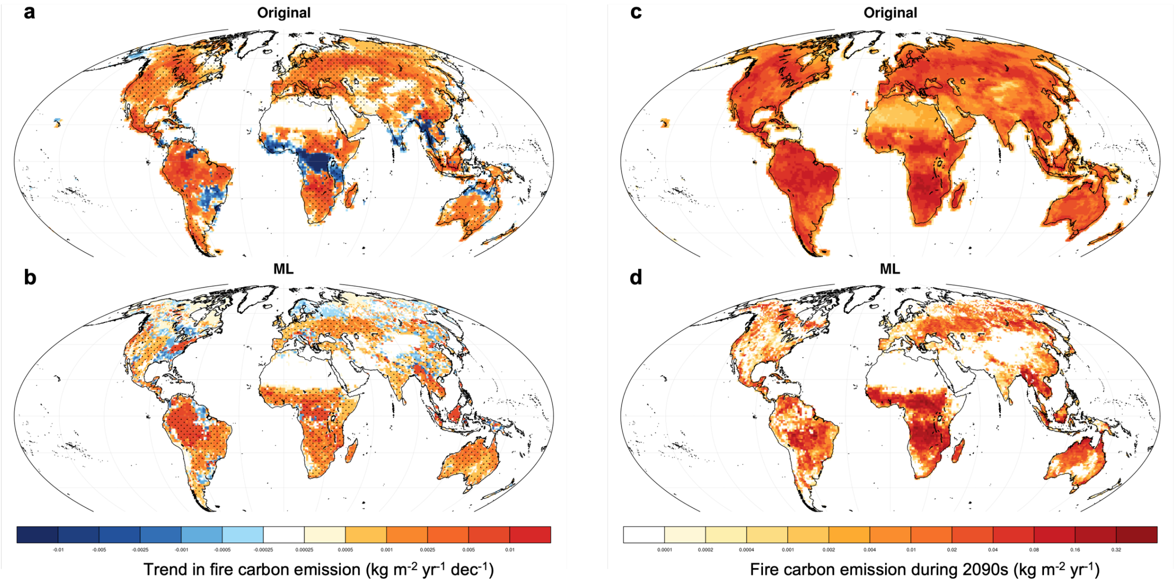

Supplementary Fig. 5: Global fire carbon emission trend and decadal mean during the 2090s projected by the default and observation-constrained ensembles. a. Multimodel mean fire carbon emission trend (kg m^{-2} yr^{-1} dec^{-1}) during the twenty-first century from the default ensemble. **b.** Multimodel mean fire carbon emission trend (kg m^{-2} yr^{-1} dec^{-1}) during the twenty-first century from the observation-constrained ensemble. **c.** Multimodel mean fire carbon emission (kg m^{-2} yr^{-1}) during the 2090s from the default ensemble. **d.** Multimode, multi-data set mean fire carbon emission (kg m^{-2} yr^{-1}) during the 2090s from the current observation-constrained ensemble.

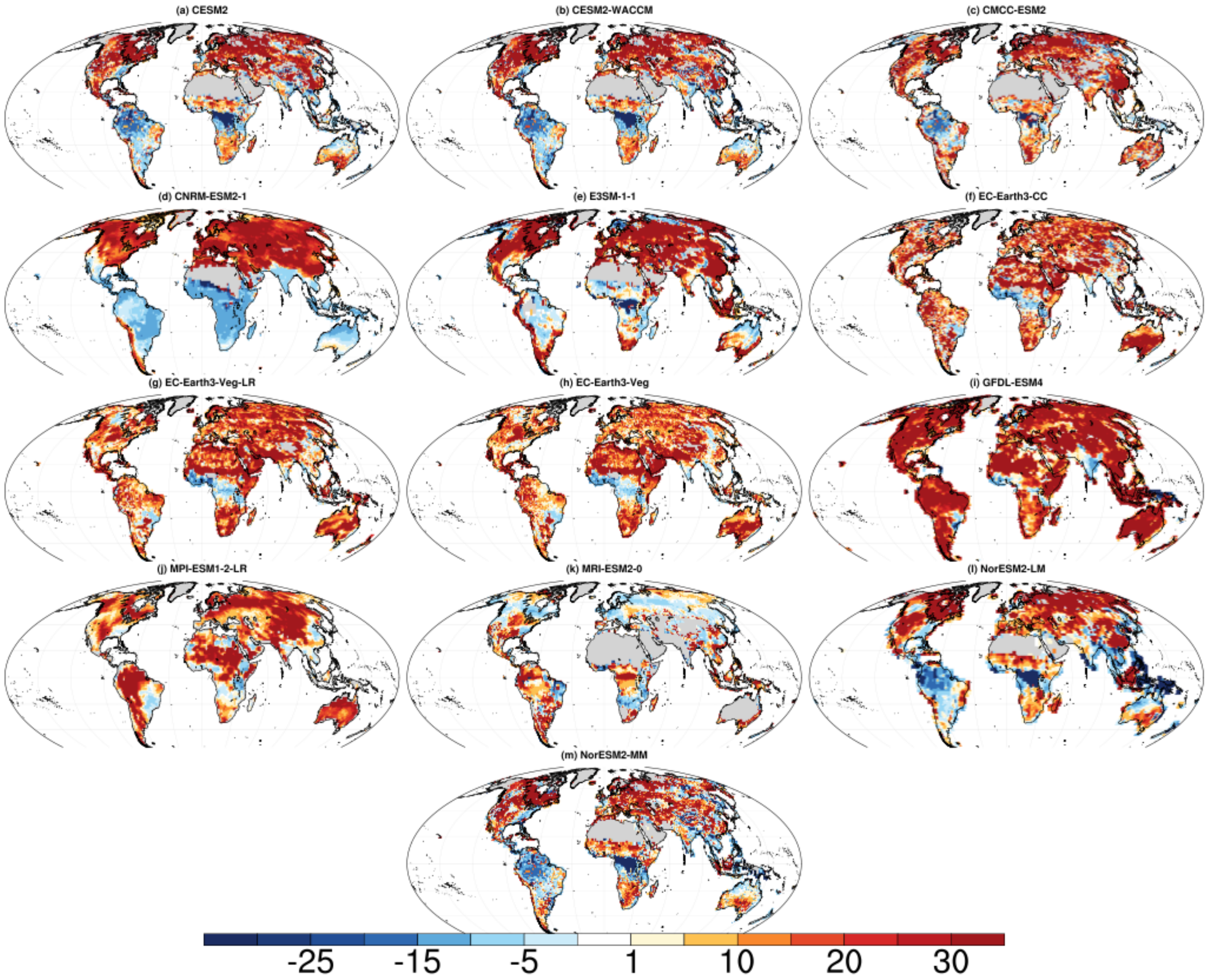

Supplementary Fig. 6: Trend in fire carbon emission during the twenty-first century simulated by the examined 13 Earth system models (ESMs). The trend is presented as relative to the historical mean fire carbon emission during the 2010s (% decade^{-1}). For models with multiple ensemble members, the ensemble mean trend is shown here.

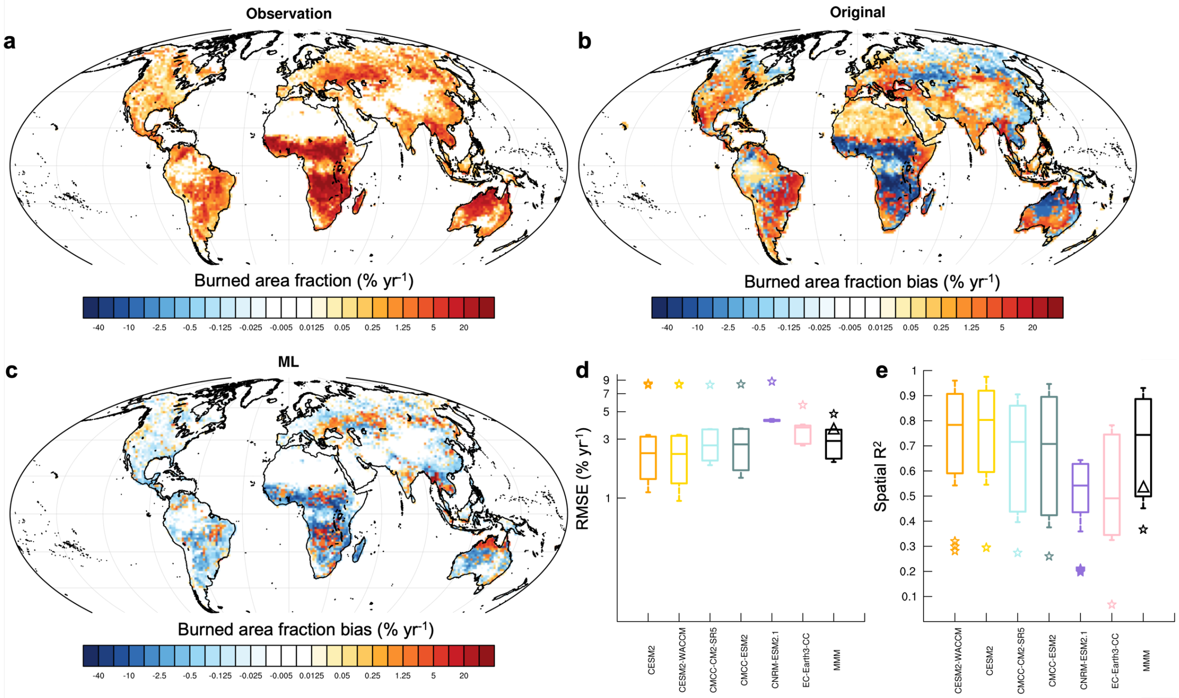

Supplementary Fig. 7: Error in historical burned area fraction during 2007–2016 from the examined six Earth system models (ESMs) before and after the observational constraint.
a. Observed mean burned area fraction, averaged across two observational data sets. **b.** Spatial distribution of the bias in original, unconstrained multimodel mean fire carbon emissions (kg m^{-2} y^{-1}). **c.** Spatial distribution of the bias in observation-constrained multimodel, multi–data set mean fire carbon emissions (kg m^{-2} yr^{-1}). **d.** Root mean square error (RMSE) between the observed, decadal mean global burned area fraction and the original (stars representing ensemble members) and observation-constrained (box plot, representing the 10th, 25th, 50th, 75th, and 90th percentiles in the multi-data-set ensemble) simulations from six ESMs and their multimodel mean. **e.** Squared spatial correlation (R^2) between the observed, decadal mean global burned area fraction and the original (stars) and observation-constrained (box plot) simulations from six ESMs and their multimodel mean. The black triangles in **d** and **e** indicate the RMSE and R^2 produced by the traditional EC approach that constrains the burned area fraction during 2007–2016 with the burned area fraction during 1997–2006.

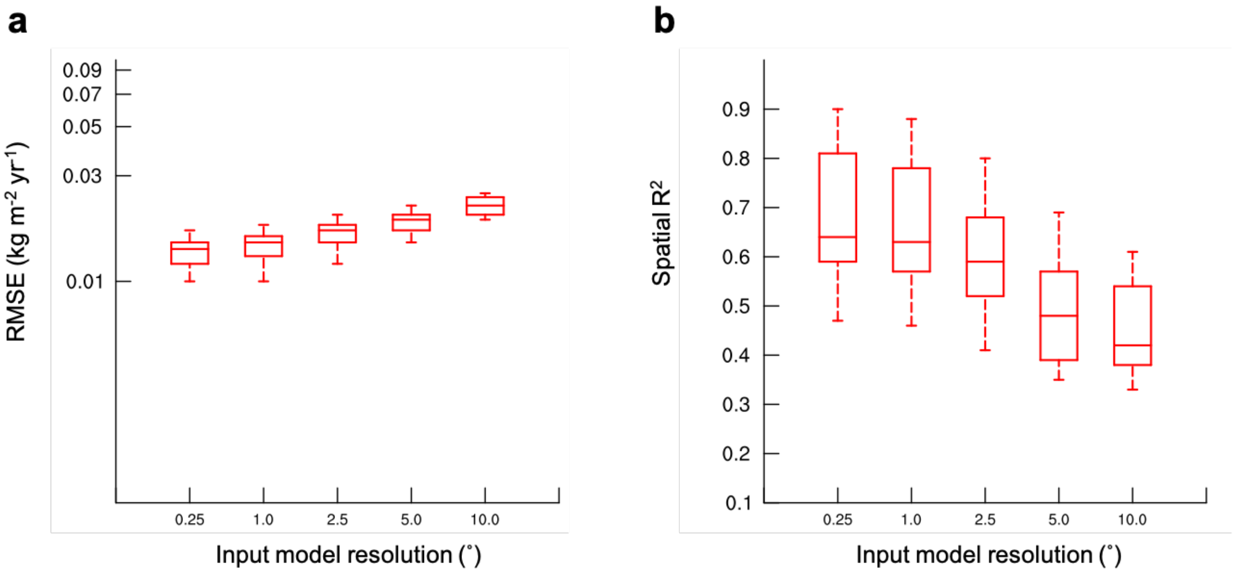

Supplementary Fig. 8: Performance of the MLT-based observation-constrained ensemble and default multimodel in the simulated fire carbon emission as a function of model resolution. The performance of the observation-constrained ensemble (red box plot, representing the 10th, 25th, 50th, 75th, and 90th percentiles of all ensemble members) and the default multimodel model mean (black star) is indicated by **a** root mean square error and **b** spatial correlation of global fire carbon emission with the average of two observational data sets. In this analysis, the input model data for the MLT-based observational constraint is first interpolated to the desired grid (0.25°, 1.0°, 2.5°, 5.0°, and 10.0° latitude/longitude) and used to train the three MLTs. Observational data sets at 0.25° resolution are subsequently fed into the fitted MLT models.

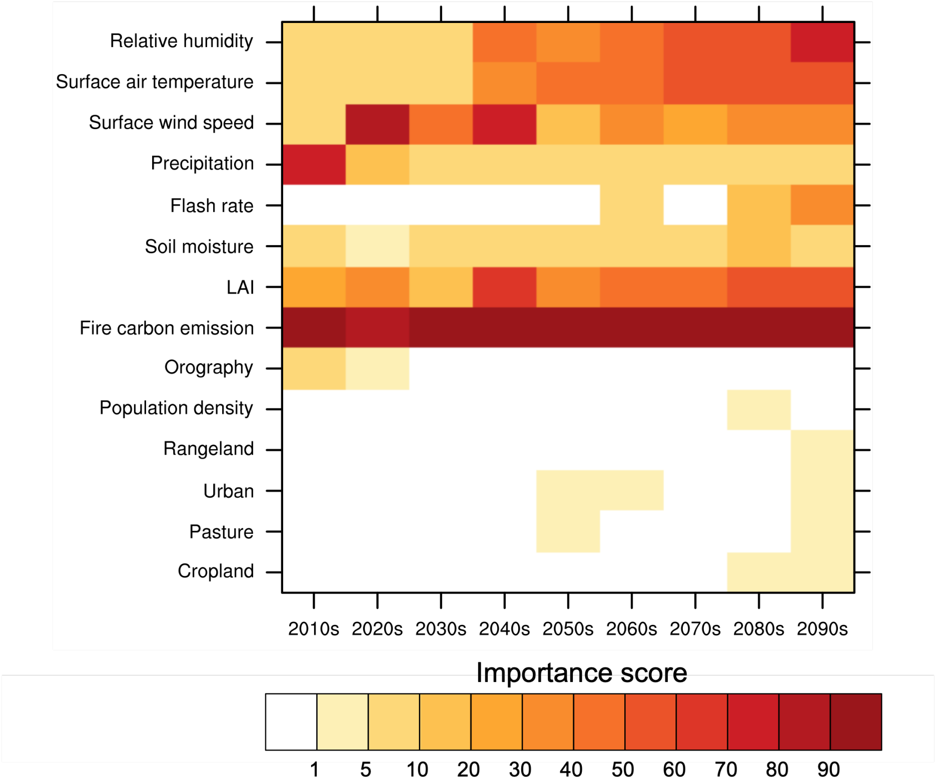

Supplementary Fig. 9: Importance of historical predictors for fire carbon emissions at different future time windows. The importance of predictors is a standard output of all machine leaning techniques (MLT). Although the calculation of importance scores varies substantially by MLT, all the importance scores qualitatively reflect relative importance of each predictor. The average importance scores from these MLT are reported here for robustness. For the atmospheric and terrestrial variables that include annual mean and monthly climatology as predictors, to account for the overall importance of a particular variable while considering the possible information overlapping contained in each month and annual mean, the importance of each variable is represented by the highest importance score among these 13 predictors (annual mean, January, February, …, December).

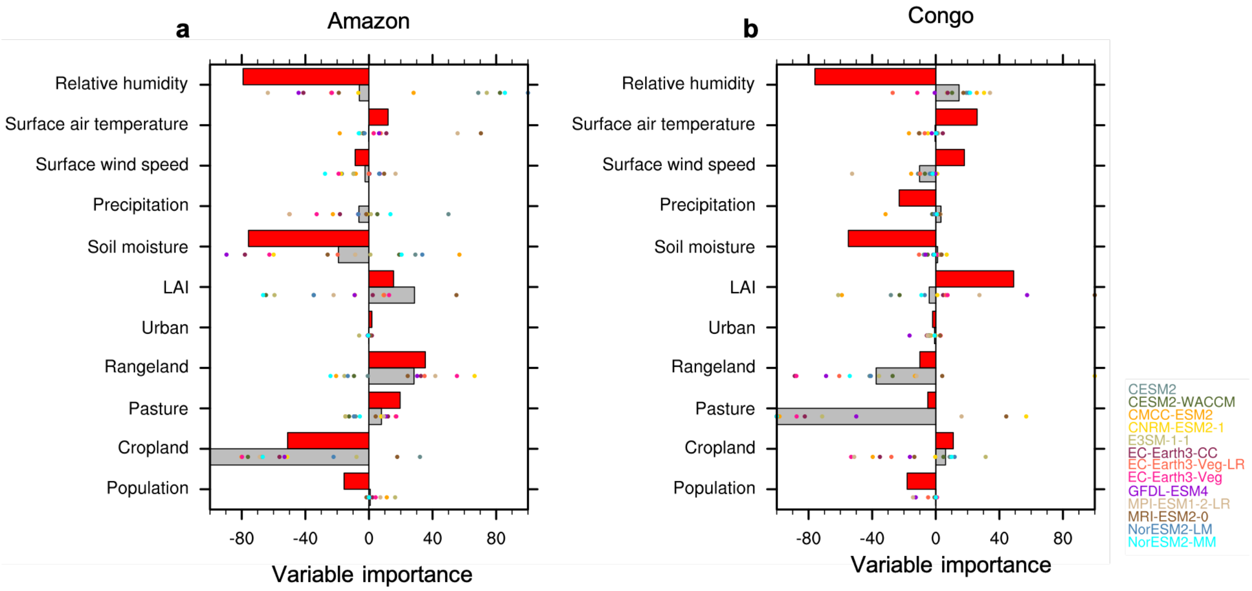

Supplementary Fig. 10: Dynamical importance of the trend in each environmental and socioeconomic driver to the spatial pattern of trend in fire carbon emissions during the twenty-first century for the tropical regions. Analyzed regions include **a.** Amazon (20°S–10°N, 80°W–40°W) and **b.** Congo (10°S–12°N, 20°W–30°E). The absolute value of the dynamical importance is qualitatively determined as the relative weight of each driver in the machine learning models for predicting the spatial distribution of the trend in fire carbon emissions (% decade^{-1}) during the twenty-first century (see Methods). The sign of the dynamical importance of each driver is assigned to the sign of spatial correlation between the projected trends in fire carbon emissions and corresponding driver. This analysis is performed for both the original (gray bars reflecting multimodel average, and individual dots representing each model) and constrained (red bars) ensembles.

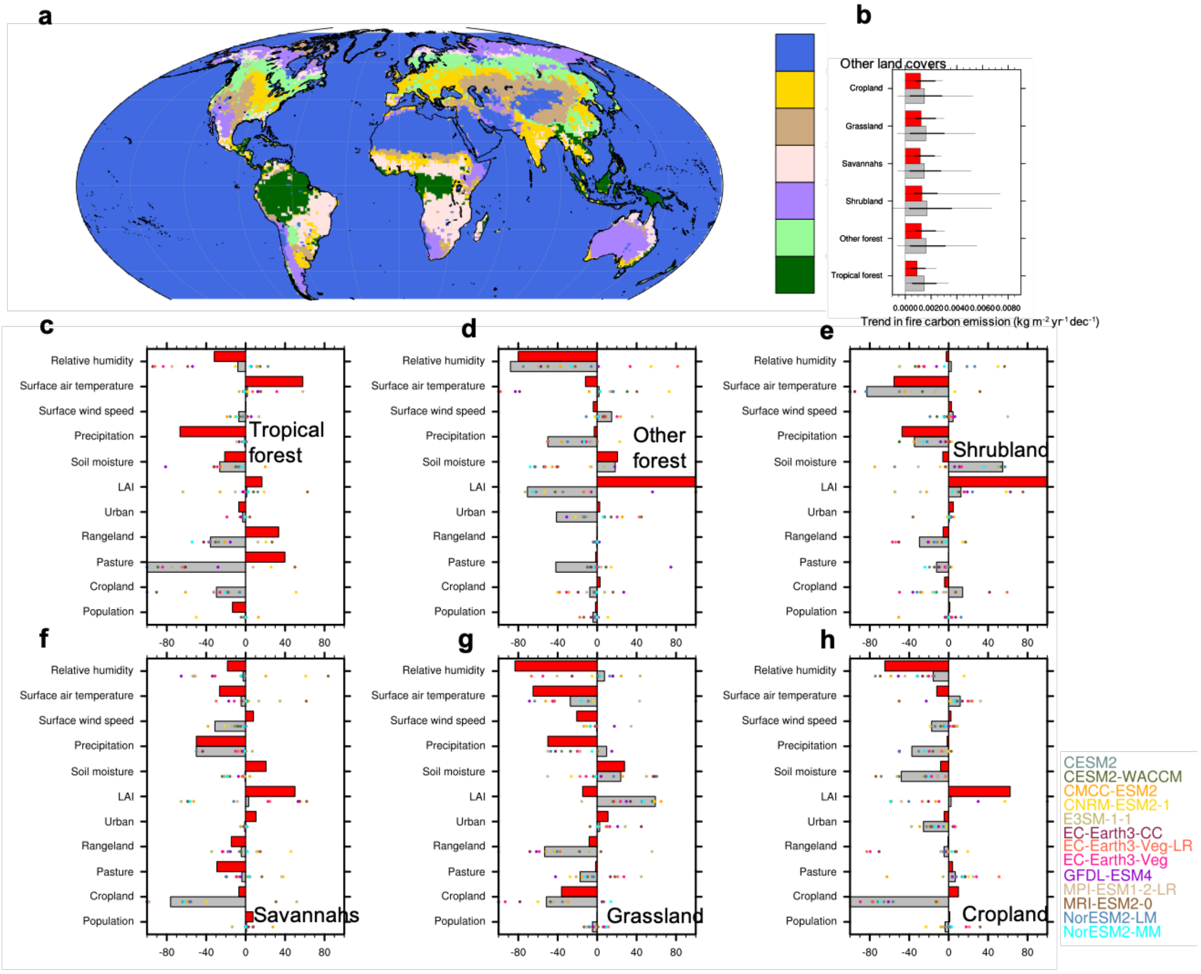

Supplementary Fig. 11: Dynamical importance of the trend in each environmental and socioeconomic driver to the spatial pattern of trend in fire carbon emissions during the twenty-first century for major land cover types. a. Primary land cover type in the historical period (2003–2020) according to the Moderate Resolution Imaging Spectroradiometer land cover product that identifies 17 classes defined by the International Geosphere-Biosphere Programme (IGBP)[37]. **b.** Box plot (10th, 25th, 50th, 75th, and 90th percentiles among all pixels with each primary land cover type) of ensemble mean trend in fire carbon emission (kg m^{-2} yr^{-1} dec^{-1}), estimated by the default (gray bars) and observation-constrained (red bars) ensemble. Tropical forest represents evergreen broadleaf forest in IGBP. Cropland represents cropland and cropland/natural vegetation mosaic. **c–h** Dynamical importance of the trend in each environmental and socioeconomic driver to the spatial pattern of trends in fire carbon emissions across **c.** tropical forest, **d.** other forest, **e.** shrubland, **f.** savannah, **g.** grassland, and **h.** cropland. The absolute value of the dynamical importance is qualitatively determined as the relative weight of each driver in the machine learning models for predicting the spatial distribution of the trend in fire carbon emissions (% $decade^{-1}$) during the twenty-first century (see Methods). The sign of the dynamical importance of each driver is assigned to the sign of spatial correlation between the projected trends in fire carbon emissions and corresponding driver. This analysis is performed for both the original (gray bars reflecting multimodel average, and individual dots representing each model) and constrained (red bars) ensembles.

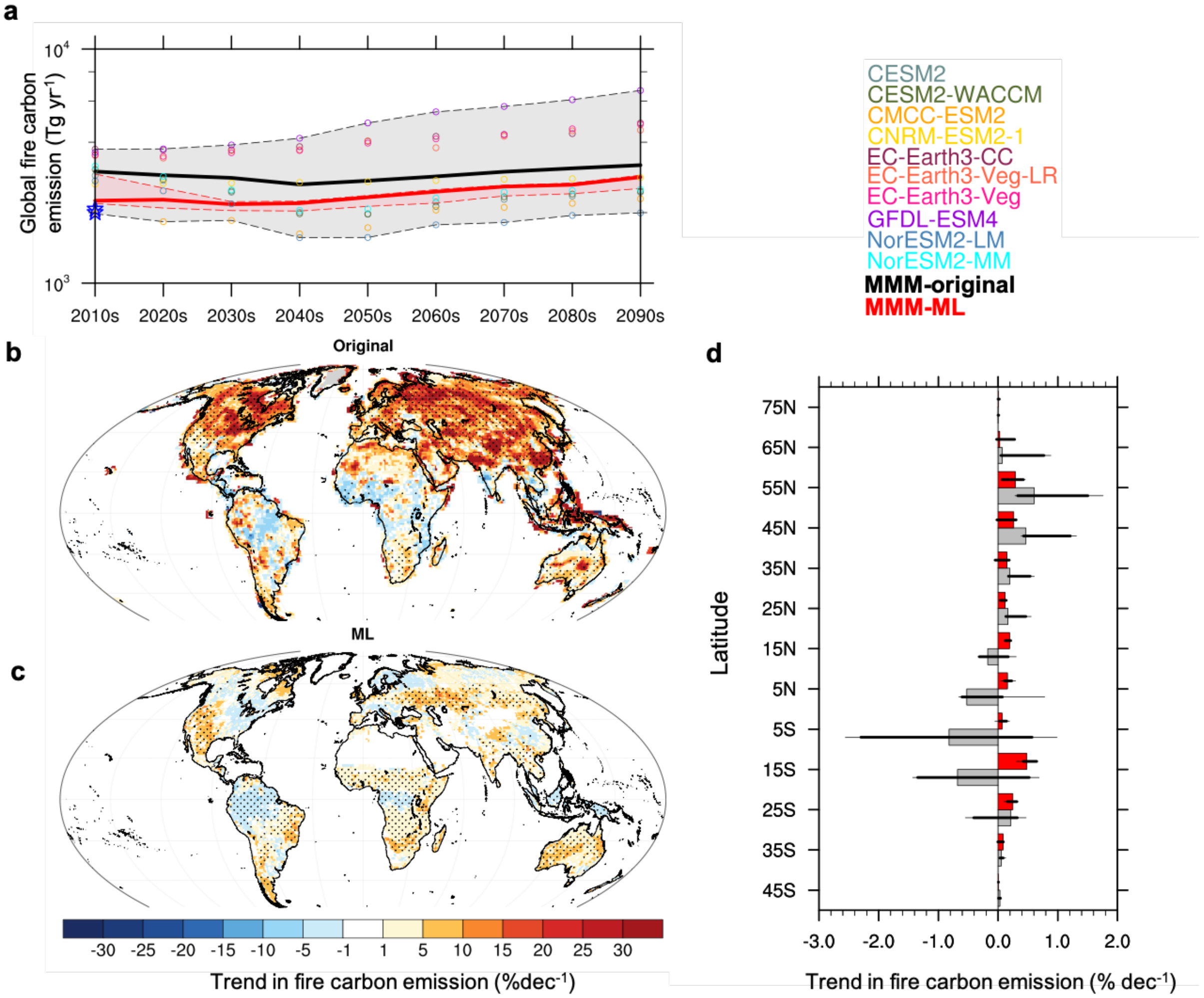

Supplementary Fig. 12: Global and latitudinal evolution of fire carbon emissions from the original and observation-constrained multimodel ensembles in SSP2-45 according to 9 CMIP6 ESMs.
a. Time series of global total fire carbon emission (Tg y^{-1}) from the 2010s to 2090s, according to the original individual ESMs (circles), their multimodel mean (thick black line), and the observation-constrained multimodel, multi–data set mean (thick red line). The pink shading with dashed boundaries indicates the 10th and 90th percentiles in the multimodel, multi–data set observation-constrained ensemble; the gray shading with dashed boundaries indicates the 10th and 90th percentiles in the original multimodel ensemble. The blue stars indicate the observed global fire carbon emission from two data sets. **b.** Unconstrained and **c.** constrained multimodel mean trends in fire carbon emission (change per decade as the percent of the historical fire carbon emission in the 2010s) from the 2010s to 2090s. Stitches indicate areas with a robust trend in fire carbon emissions, with a consistent sign of trend among at least 80% of the ensemble members. **d.** Trend in the total fire carbon emission (change per decade as the percentage of the historical fire carbon emission in the 2010s per decade) from each 10° zonal band during the 2010s to 2090s, according to the original (multimodel mean: gray bars; 25th–75th percentiles: thick horizontal lines; 10th–90th percentiles: thin horizontal lines) and observation-constrained (multimodel mean: red bars; 25th–75th percentiles: thick horizontal lines; 10th–90th percentiles: thin horizontal lines) ESM simulations.

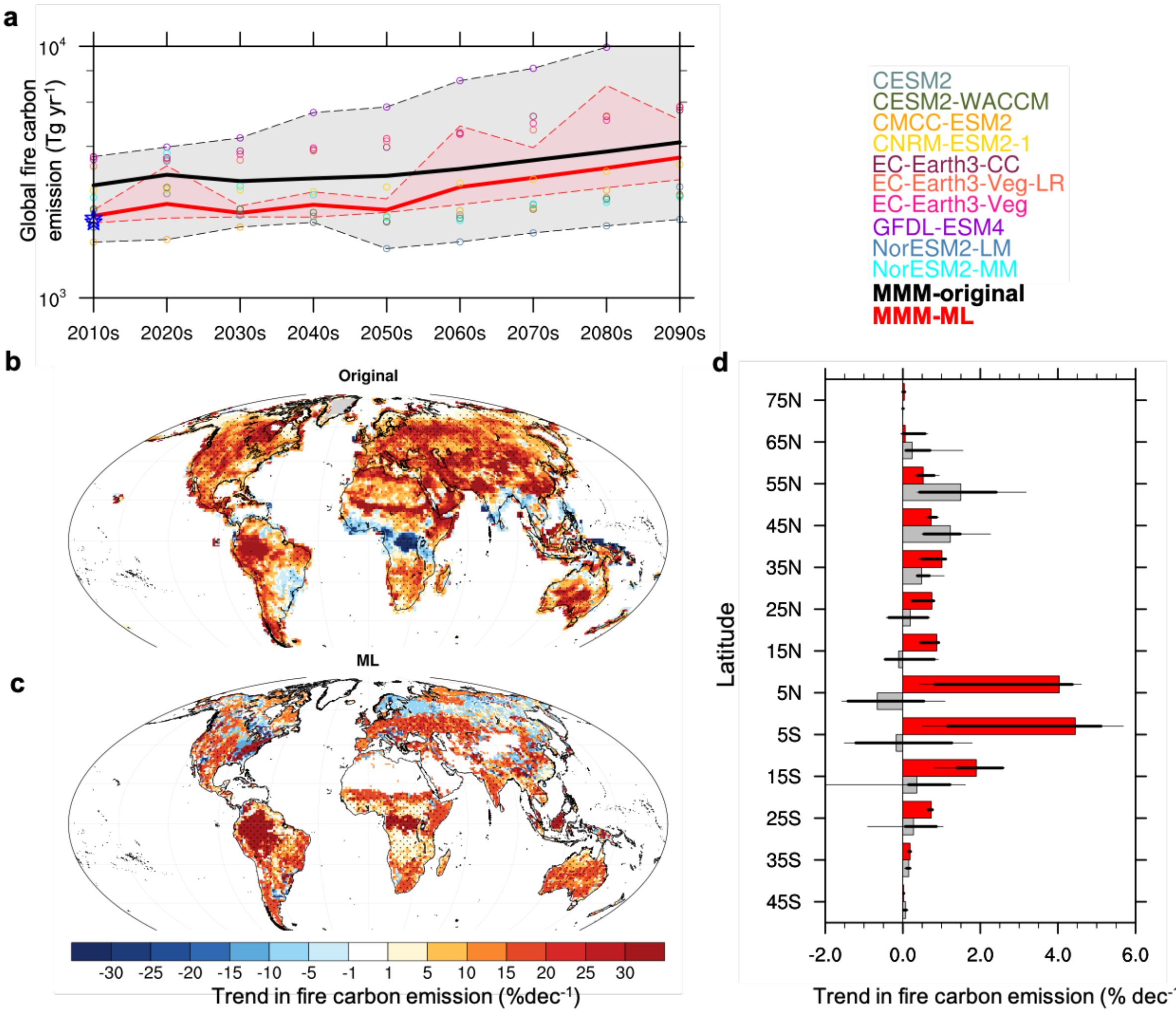

Supplementary Fig. 13: Global and latitudinal evolution of fire carbon emissions from the original and observation-constrained multimodel ensembles in SSP5-85 according to the same nine CMIP6 ESMs that provide SSP2-45 simulations in Extended Data Fig. 12. a. Time series of global total fire carbon emission (Tg y^{-1}) from the 2010s to 2090s, according to the original individual ESMs (circles), their multimodel mean (black thick line), and the observation-constrained multimodel, multi–data set mean (red thick line). The pink shading with dashed boundaries indicates the 10th and 90th percentiles in the multimodel, multi–data set observation-constrained ensemble; the gray shading with dashed boundaries indicates the 10th and 90th percentiles in the original multimodel ensemble. The blue stars indicate the observed global fire carbon emission from two data sets. **b.** Unconstrained and **c.** constrained multimodel mean trend in fire carbon emission (change per decade as the percentage of the historical fire carbon emission in the 2010s) from the 2010s to 2090s. Stitches indicate areas with a robust trend in fire carbon emission, with a consistent sign of trend among at least 80% of the ensemble members. **d.** Trend in the total fire carbon emission (change per decade as the percentage of the historical fire carbon emission in the 2010s) from each 10° zonal band during the 2010s to 2090s, according to the original (multimodel mean: gray bars; 25th–75th percentiles: thick horizontal lines; 10th–90th percentiles: thin horizontal lines) and observation-constrained (multimodel mean: red bars; 25th–75th percentiles: thick horizontal lines; 10th–90th percentiles: thin horizontal lines) ESM simulations.

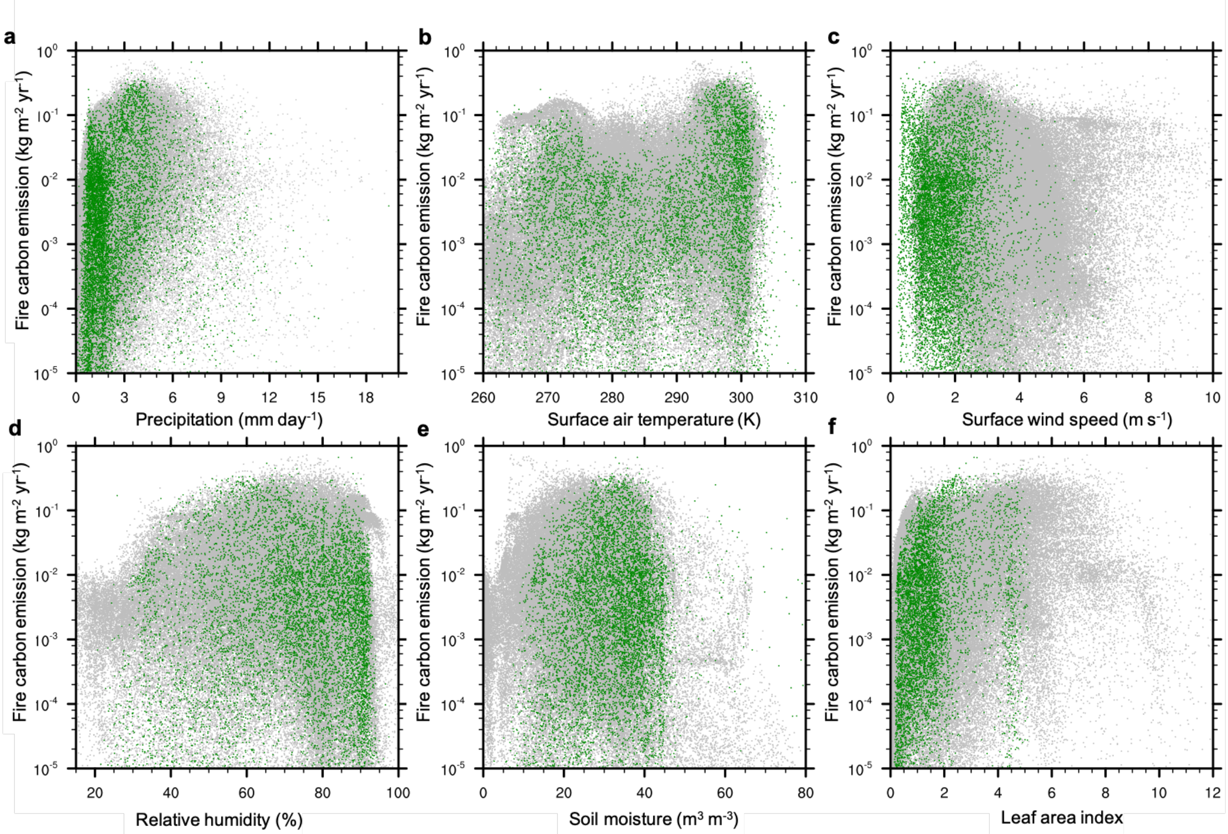

Supplementary Fig. 14: Data space covered by the observations and historical simulation of the examined 13 CMIP6 ESMs. The joint data space is demonstrated by two-variable scatterplots of fire carbon emission versus **a.** precipitation, **b.** surface air temperature, **c.** surface wind speed, **d.** relative humidity, **e.** soil moisture, and **f.** leaf area index from the global spatial sample (11,325 pixels per model). The gray dots (11,325 pixels model^{-1} × 13 models) are from ESM historical simulation, and green dots are from observational data sets. If multiple observational data sets are available, their average is shown.

References

1. Danabasoglu, G. *et al.* The Community Earth System Model Version 2 (CESM2). *J. Adv. Model. Earth Syst.* **12**, 1–35 (2020).
2. Li, F., Levis, S. & Ward, D. S. Quantifying the role of fire in the Earth system - Part 1: Improved global fire modeling in the Community Earth System Model (CESM1). *Biogeosciences* **10**, 2293–2314 (2013).
3. Li, F., Zeng, X. D. & Levis, S. A process-based fire parameterization of intermediate complexity in a dynamic global vegetation model. *Biogeosciences* **9**, 2761–2780 (2012).
4. Cherchi, A. *et al.* Global Mean Climate and Main Patterns of Variability in the CMCC-CM2 Coupled Model. *J. Adv. Model. Earth Syst.* **11**, 185–209 (2019).
5. Séférian, R. *et al.* Evaluation of CNRM Earth System Model, CNRM-ESM2-1: Role of Earth System Processes in Present-Day and Future Climate. *J. Adv. Model. Earth Syst.* **11**, 4182–4227 (2019).
6. Golaz, J. C. *et al.* The DOE E3SM Coupled Model Version 1: Overview and Evaluation at Standard Resolution. *J. Adv. Model. Earth Syst.* **11**, 2089–2129 (2019).
7. Döscher, R. *et al.* The EC-Earth3 Earth System Model for the Climate Model Intercomparison Project 6. *Geosci. Model Dev. Discuss.* 1–90 (2021). doi:10.5194/gmd-2020-446
8. Dunne, J. P. *et al.* The GFDL Earth System Model version 4.1 (GFDL-ESM4.1): Model description and simulation characteristics. *J. Adv. Model. Earth Syst.* **submitted**, (2020).
9. Rabin, S. S. *et al.* A fire model with distinct crop, pasture, and non-agricultural burning: Use of new data and a model-fitting algorithm for FINAL.1. *Geosci. Model Dev.* **11**, 815–842 (2018).
10. Mauritsen, T. *et al.* Developments in the MPI-M Earth System Model version 1.2 (MPI-ESM1.2) and Its Response to Increasing CO2. *J. Adv. Model. Earth Syst.* **11**, 998–1038 (2019).
11. Yukimoto, S. *et al.* The meteorological research institute Earth system model version 2.0, MRI-ESM2.0: Description and basic evaluation of the physical component. *J. Meteorol. Soc. Japan* **97**, 931–965 (2019).
12. Seland, Ø. *et al. Overview of the Norwegian Earth System Model (NorESM2) and key climate response of CMIP6 DECK, historical, and scenario simulations*. *Geoscientific Model Development* **13**, (2020).
13. Van Der Werf, G. R. *et al.* Global fire emissions and the contribution of deforestation, savanna, forest, agricultural, and peat fires (1997-2009). *Atmos. Chem. Phys.* **10**, 11707–11735 (2010).
14. Kaiser, J. W. *et al.* Biomass burning emissions estimated with a global fire assimilation system based on observed fire radiative power. *Biogeosciences* **9**, 527–554 (2012).
15. Zhu, Z. *et al.* Global data sets of vegetation leaf area index (LAI) 3g and Fraction of Photosynthetically Active Radiation (FPAR) 3g derived from Global Inventory Modeling and Mapping Studies (GIMMS) Normalized Difference Vegetation Index (NDVI3g) for the period 1981 to. *Remote Sens.* **5**, 927–948 (2013).
16. Liu, Y., Liu, R. & Chen, J. M. Retrospective retrieval of long-term consistent global leaf area index (1981-2011) from combined AVHRR and MODIS data. *J. Geophys. Res. G Biogeosciences* **117**, (2012).
17. Vermote, E. & NOAA CDR Program. NOAA Climate Data Record (CDR) of AVHRR Leaf Area Index (LAI) and Fraction of Absorbed Photosynthetically Active Radiation (FAPAR), Version 5. doi:https://doi.org/10.7289/V5TT4P69
18. Martens, B. *et al.* GLEAM v3: Satellite-based land evaporation and root-zone soil moisture. *Geosci. Model Dev.* **10**, 1903–1925 (2017).
19. Dee, D. P. *et al.* The ERA-Interim reanalysis: Configuration and performance of the data assimilation system. *Q. J. R. Meteorol. Soc.* **137**, 553–597 (2011).
20. Wang, Y. *et al.* Development of observation-based global multilayer soil moisture products for 1970 to 2016. *Earth Syst. Sci. Data* **13**, 4385–4405 (2021).
21. Forkel, M. *et al.* A data-driven approach to identify controls on global fire activity from satellite and climate observations (SOFIA V1). *Geosci. Model Dev.* **10**, 4443–4476 (2017).

22. Di Giuseppe, F. *et al.* The potential predictability of fire danger provided by numerical weather prediction. *J. Appl. Meteorol. Climatol.* **55**, 2469–2491 (2016).
23. Matsuura, K. & Willmott, C. J. Terrestrial Air Temperature: 1900-2017 Gridded Monthly Time Series. (2018). Available at: http://climate.geog.udel.edu/~climate/html_pages/Global2017/README.GlobalTsT2017.html.
24. Harris, I., Jones, P. D., Osborn, T. J. & Lister, D. H. Updated high-resolution grids of monthly climatic observations - the CRU TS3.10 Dataset. *Int. J. Climatol.* **34**, 623–642 (2014).
25. Matsuura, K. & Willmott, C. J. Terrestrial Precipitation: 1900-2017 Gridded Monthly Time Series. (2018). Available at: http://climate.geog.udel.edu/~climate/html_pages/Global2017/README.GlobalTsP2017.html.
26. Schneider, U. *et al.* GPCC's new land surface precipitation climatology based on quality-controlled in situ data and its role in quantifying the global water cycle. *Theor. Appl. Climatol.* **115**, 15–40 (2014).
27. Rienecker, M. M. *et al.* MERRA: NASA's Modern-Era Retrospective Analysis for Research and Applications. *J. Clim.* **24**, 3624–3648 (2011).
28. Hersbach, H. *et al.* The ERA5 global reanalysis. *Q. J. R. Meteorol. Soc.* **146**, 1999–2049 (2020).
29. Taylor, S. W., Woolford, D. G., Dean, C. B. & Martell, D. L. Wildfire prediction to inform fire management: Statistical science challenges. *Stat. Sci.* **28**, 586–615 (2013).
30. Cecil, D. J., Buechler, D. E. & Blakeslee, R. J. Gridded lightning climatology from TRMM-LIS and OTD: Dataset description. *Atmos. Res.* **135–136**, 404–414 (2014).
31. Sharples, J. J. *et al.* Natural hazards in Australia: extreme bushfire. *Clim. Change* **139**, 85–99 (2016).
32. Di Virgilio, G. *et al.* Climate Change Increases the Potential for Extreme Wildfires. *Geophys. Res. Lett.* **46**, 8517–8526 (2019).
33. Abram, N. J. *et al.* Connections of climate change and variability to large and extreme forest fires in southeast Australia. *Commun. Earth Environ.* **2**, (2021).
34. National Geophysical Data Center/NESDIS/NOAA/U.S. Department of Commerce. TerrainBase, Global 5 Arc-minute Ocean Depth and Land Elevation from the US National Geophysical Data Center (NGDC). (1995). doi:10.5065/E08M-4482
35. Hurtt, G. C. *et al.* Harmonization of land-use scenarios for the period 1500-2100: 600 years of global gridded annual land-use transitions, wood harvest, and resulting secondary lands. *Clim. Change* **109**, 117–161 (2011).
36. Goldewijk, K. K., Beusen, A., Doelman, J. & Stehfest, E. Anthropogenic land use estimates for the Holocene - HYDE 3.2. *Earth Syst. Sci. Data* **9**, 927–953 (2017).
37. Friedl, M. A. & Sulla-Menashe, D. *User Guide to Collection 6 MODIS Land Cover (MCD12Q1 and MCD12C1) Product*. **6**, (2018).
